# A proposal for clinical trials of COVID-19 treatment using homo-harringtonine

**DOI:** 10.1093/nsr/nwaa257

**Published:** 2020-10-12

**Authors:** Hai-Jun Wen, Feng-Liang Liu, Ming-Xing Huang, Rong-Hua Luo, Wen-Bin He, Jing Feng, Fang-Liang Chen, Qi-Chun Cai, Hua-Juan Ma, Zi-Feng Yang, Xi Zhou, You Shang, Xue-Mei Lyu, Ding-Yu Zhang, Fei Xiao, Hong Shan, Jian-Xing He, Yong-Tang Zheng, Chung-I Wu

**Affiliations:** State Key Laboratory of Biocontrol, School of Life Sciences, Sun Yat-sen University, China; Key Laboratory of Animal Models and Human Disease Mechanisms of Chinese Academy of Sciences/Key Laboratory of Bioactive Peptides of Yunnan Province, Kunming Institute of Zoology, Chinese Academy of Sciences, China; Kunming National High-Level Bio-Safety Research Center for Non-Human Primates, Center for Biosafety Mega-Science, Kunming Institute of Zoology, Chinese Academy of Sciences, China; Department of Infectious Diseases, The Fifth Affiliated Hospital, Sun Yat-sen University, China; Key Laboratory of Animal Models and Human Disease Mechanisms of Chinese Academy of Sciences/Key Laboratory of Bioactive Peptides of Yunnan Province, Kunming Institute of Zoology, Chinese Academy of Sciences, China; Kunming National High-Level Bio-Safety Research Center for Non-Human Primates, Center for Biosafety Mega-Science, Kunming Institute of Zoology, Chinese Academy of Sciences, China; State Key Laboratory of Genetic Resources and Evolution, Kunming Institute of Zoology, Chinese Academy of Sciences, China; Center for Excellence in Animal Evolution and Genetics, Chinese Academy of Sciences, China; State Key Laboratory of Genetic Resources and Evolution, Kunming Institute of Zoology, Chinese Academy of Sciences, China; Center for Excellence in Animal Evolution and Genetics, Chinese Academy of Sciences, China; Kunming Police Dog Base of the Ministry of Public Security, China; Cancer Center, Clifford Hospital, Jinan University, China; Cancer Center, Clifford Hospital, Jinan University, China; National Clinical Research Center for Respiratory Disease, Guangzhou Institute of Respiratory Health, First Affiliated Hospital of Guangzhou Medical University, State Key Laboratory of Respiratory Disease (Guangzhou Medical University), China; Faculty of Chinese Medicine, Macau University of Science and Technology, China; Wuhan Institute of Virology, Center for Biosafety Mega-Science, Chinese Academy of Sciences, China; Center for Translational Medicine, Wuhan Jinyintan Hospital, China; Joint Laboratory of Infectious Diseases and Health, Wuhan Institute of Virology and Wuhan Jinyintan Hospital, China; Joint Laboratory of Infectious Diseases and Health, Wuhan Institute of Virology and Wuhan Jinyintan Hospital, China; Tongji Medical College, Huazhong University of Science and Technology, China; State Key Laboratory of Genetic Resources and Evolution, Kunming Institute of Zoology, Chinese Academy of Sciences, China; Center for Excellence in Animal Evolution and Genetics, Chinese Academy of Sciences, China; Center for Translational Medicine, Wuhan Jinyintan Hospital, China; Joint Laboratory of Infectious Diseases and Health, Wuhan Institute of Virology and Wuhan Jinyintan Hospital, China; Department of Infectious Diseases, The Fifth Affiliated Hospital, Sun Yat-sen University, China; Guangdong Provincial Key Laboratory of Biomedical Imaging and Guangdong Provincial Engineering Research Center of Molecular Imaging, The Fifth Affiliated Hospital, Sun Yat-sen University, China; Guangdong Provincial Key Laboratory of Biomedical Imaging and Guangdong Provincial Engineering Research Center of Molecular Imaging, The Fifth Affiliated Hospital, Sun Yat-sen University, China; Center for Interventional Medical, The Fifth Affiliated Hospital, Sun Yat-sen University, China; National Clinical Research Center for Respiratory Disease, Guangzhou Institute of Respiratory Health, First Affiliated Hospital of Guangzhou Medical University, State Key Laboratory of Respiratory Disease (Guangzhou Medical University), China; Key Laboratory of Animal Models and Human Disease Mechanisms of Chinese Academy of Sciences/Key Laboratory of Bioactive Peptides of Yunnan Province, Kunming Institute of Zoology, Chinese Academy of Sciences, China; Kunming National High-Level Bio-Safety Research Center for Non-Human Primates, Center for Biosafety Mega-Science, Kunming Institute of Zoology, Chinese Academy of Sciences, China; State Key Laboratory of Biocontrol, School of Life Sciences, Sun Yat-sen University, China

Dear editor,

A scheme for treating COVID-19 was published in *NSR* in April [1] based on blocking the translation of very large viral proteins. Since then, additional supporting data have greatly strengthened the proposal. The drug, HHT (homo-harringtonine, or omacetaxine), is readily available and inexpensive as it has been approved since 2012 for treating leukemia. The *in vitro* and *in vivo* data clearly justify the planning of clinical trials. Given the specific mechanism, we believe a trial of modest scale would be sufficient to prove, or disprove, the efficacy of the treatment scheme. Such trials, however, are only feasible in regions with many new infections.

This letter, commenting on Wu and Wen [1], has an expanded authorship that includes physicians with first-hand experience in treating COVID-19 patients and virologists with recent publications on SARS-CoV-2. We wish to bring this proposal to the attention of the global community as there are few new cases in China for such trials.

## THE ORIGINAL PROPOSAL

On 15 April 2020, we published a proposal [1] in *NSR* on a potential treatment of COVID-19. The proposal is based on the heightened activities of protein translation in two types of aberrant mammalian cells—cancer cells and virus-infected cells. The FDA approved drug, HHT, has been known to disrupt the elongation step of protein translation with very high efficiency [2–4]. HHT, approved for treating leukemia since 2012 [5], is probably the most powerful drug known to block protein translation.

There are several advantages to HHT over other drugs that block protein translation [6,7]. The details can be found in Wu and Wen [1] and a brief summary is provided below. (i) The making of a ‘super-protein’ that will then yield 16 non-structural proteins by protease cleavage could be the Achilles heel of the coronaviruses including SARS-CoV-2. Since very few human proteins can rival this super-protein in size, it should be possible to preferentially disrupt the translation of viral proteins with minimal damage to the un-infected human cells. (ii) Because HHT has been approved for clinical use since 2012, the safe dosage and toxicity effect are well understood. (iii) The HHT dose required for suppressing virus proliferation is in the range of nano-molar concentration whereas the toxicity to human cells requires a concentration that is at least 30 times higher. (iv) This low dose may permit several convenient means of drug delivery (see below).

Until very recently, glucocorticoids were the only intervention that reduced COVID-19-related death compared with the standard care. Three other drugs with modest efficacy are currently in use: hydroxychloroquine, remdesivir and lopinavir-ritonavir [8], in addition to the many published schemes [9–16]. Given its well-known efficacy and safety, HHT deserves to enter clinical trials, along with other drugs announced to have the potential to block viral protein translation [17].

## THE NEW EVIDENCE—*IN VITRO* AND *IN VIVO* EXPERIMENTS

Although there was a sense of urgency for new treatment proposals, it was also apparent that the HHT scheme would need additional supports, which are provided here.

### *In vitro* efficacy

**Figure 1.**
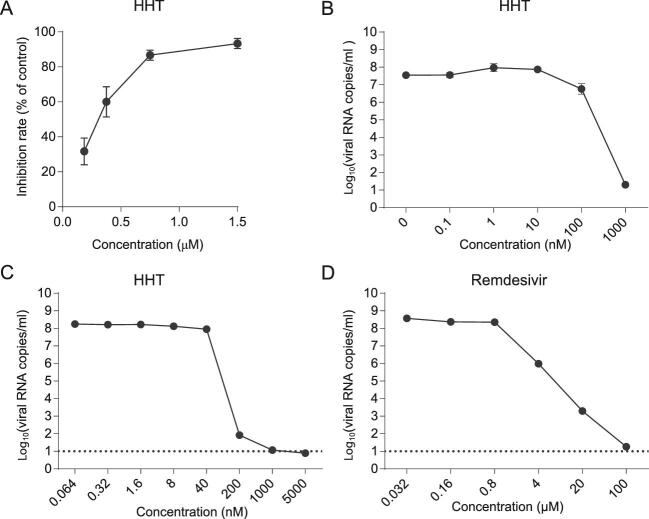
HHT inhibition of SARS-CoV-2 replication *in vitro* as performed by various laboratories, including (A) State Key Laboratory of Respiratory Disease (Guangzhou Medical University); (B) Harbin Veterinary Research Institute; and (C) Kunming Institute of Zoology. (D) For a comparison, the inhibitory effect of remdesivir on SARS-CoV-2 was also assayed in Kunming Institute of Zoology. The horizontal dashed line in (C) and (D) indicates complete inhibition against SARS-CoV-2. At 1 μM, HHT can clear the virus whereas remdesivir would require 20–100 μM to have the same effect.

The original proposal cited the efficacy of HHT on coronaviruses in general. We now provide new data on SARS-CoV-2. The IC_50_ is between <100–350 nM depending on the conditions of the experiments and the lot of HHT used (Fig. 1A–C). It is also clear that, at 1 μM, the killing of the viruses is complete. As a comparison, remdesivir needs >20 μM to achieve a similar effect (Fig. 1D). Hence, the efficacy of HHT in clearing SARS-CoV-2 is in line with its efficacy against other coronaviruses [18–21]. In a recent study, Choy *et al.* tested 16 compounds for SARS-CoV-2 inhibition and HHT stands out as well [22]. (We notice that the concentration needed to achieve EC_50_ is often much higher in Choy *et al.*’s data than in other experiments by 10–30 fold, although the relative efficacy among drugs is consistent with others.)

As the half-cytotoxic concentration (CC_50_) of HHT in mammalian cells usually exceeds 10 uM [18,19,21], HHT has a selectivity index (SI = CC_50_/IC_50_) >30. Taken together, these results confirm the promises of HHT in treating COVID-19.

### *In vivo* efficacy

In a preliminary experiment, we infected ACE2 humanized mice with SARS-CoV-2. This inbred mice model was generated by integrating CRISPR-Cas9 and tetraploid complementation. The infected mice were either untreated as the control or treated by intraperitoneal injection of 40 μg HHT in 100 μl normal saline. The first dose is administered 2 hours before the virus challenge. Since HHT inhibits viral replication but does not block virus entry, the lead time of 2 hours should be sufficient for drug

diffusion into the cells. HHT is administered daily for 3 days.

On the third day post infection (d.p.i), mice were sacrificed and SARS-CoV-2 genomic RNA in lung tissues was measured by qRT-PCR. Among the three untreated mice, we obtained from 15 samples (five lobes of the lung from each mouse) the viral load of each sample, classified as ND (non-detectable), background (<10^2^ copies/μg total RNA) or high load (10^5^–10^8^). We should note that the infected mice, if untreated, would start to clear the viral load starting on the fourth day. The distribution of viral load, summarized as [ND, Low, High], was [3, 6, 6] for the control. In contrast, the distribution for the three HHT-treated mice was [9, 6, 0]. Although the sample size was small, HHT did repress the viral load to the background level in all 15 samples whereas the load was high in 6 of the 15 untreated samples. Given all the evidence, we believe that clinical trials should be the next step as suggested below.

## PROPOSED CLINICAL TRIAL—I. THE PRE-TREATMENT

From the standpoint of public health, the goal is to stop the spread of the virus and the progression of the infection, *before* the infection is confirmed. Here, we propose a two-step scheme aimed at the clearance of the viral load in the early phases of COVID infection. Late phases of infection with severe organ damage are not the target stages. For individuals who are not yet confirmed for infection but have reason to suspect the possibility (such as close contact with positive cases), we now recommend a pre-treatment procedure as follows.

First, as shown by Wölfel *et al.* [23] and He *et al.* [24], SARS-CoV-2 viruses may spend the first four days, on average, in the throat and nasal passage before they enter the lower respiratory tract. Second, the World Health Organization guideline [25] has shown that SARS-CoV-2 virus cannot survive for more than 30 seconds in 30% alcohol. Hence, a pre-treatment by liquid with an alcohol content of above 30% (as in many liquor beverages), gargling 2–3 times a day each time for 30 seconds, might be effective in substantially arresting disease progression as well as inhibiting the spread of the virus to others.

There have indeed been reports of alcohol's efficacy in reducing the viral load during COVID-19 [26]. If the disease symptoms do progress, then the HHT treatment would be the next step.

## PROPOSED CLINICAL TRIAL—II. THE HHT DELIVERY

If the pre-treatment fails to clear the virus, an option would be the standard delivery of HHT by intravenous injection, which lasts at least 3 hours each day in a course of 4–6 days in treating leukemia over several courses.

- Intravenous injection: the safety level of HHT by intravenous injection is well-known.
  - *In vitro* dosage: Human HEK-293T cells can survive the 20 μM treatment for >3 hours or 5 μM for >24 hours. Indeed, 5 μM is far higher than necessary for suppressing SARS-CoV-2.
  - Dosage for the HHT treatment of leukemia: 1.5 mg/m^2^ (∼2.5 mg/[60 kg person] or 100 nM for adult patients) with daily continuous intravenous infusion for 3–6 hours up to 28 days.
  - It has been reported that the maximum tolerated and therapeutically effective dose of HHT for treating adult leukemia patients is 4 mg daily for 14 days [27]. The treatment duration for COVID-19 is likely to be much shorter.
- Nebulization: an alternative delivery would be by nebulization, although safety with regard to humans has to be evaluated. There are reasons for preferring nebulization to intravenous injection in reducing the viral load and stopping the progression of symptoms. With nebulization, the drug concentration should be higher in the lungs than in other tissues, but probably for no more than a few hours after nebulization [28]. The drug would then be distributed through blood circulation as in intravenous injection.

In previous reports, HHT delivered by subcutaneous injection can only achieve a systemic concentration of 50–60 nM while intravenous injection may achieve a higher concentration of 100–200 nM [29,30]. The HHT concentration achievable by intravenous injection is very close to the *in vitro* IC_50_ at 100–300 nM, thus leaving almost no margin for error. Furthermore, nebulization can be more easily administered by the patients themselves than intravenous injection when multiple applications per day are called for. A caveat is that aerosols generated during nebulization should be strictly regulated to avoid viral spread.

Since the toxicity by nebulization, especially to the lungs, is not known, we have assessed the effect of HHT nebulization on two dogs, each weighing ∼6 kg. Dogs may be the only animal model on which nebulization can be applied. The dosage was ramped up from 0.2 mg to 0.4, 0.6, 0.8 and 1 mg per day. Each dosage was applied in two successive days with the whole treatment lasting 10 days. Each dog was monitored by daily physical assessment, complete blood count and chemistry profile, as well as a chest X-ray examination every two days. No toxicological changes related to the treatment were observed. Since the nebulization treatment in humans can be delivered evenly in the course of a day, 1 mg of HHT for humans should be well tolerated for several days.

### Recommended parameters

If the goal is to sustain a higher concentration of HHT in the lungs, we would recommend several episodes of nebulization per day (see also the next section). Based on the dosage used in treating leukemia of a person weighing 60 kg, we suggest 1 mg per day in 15 ml saline by nebulization five times a day at 4-hour intervals, allowing 8 hours sleep. Each episode would be 15 minutes to nebulize 3 ml of solution. The total dose may be gradually ramped up to 4 mg per day if safety permits.

The recommendation is based on our knowledge of sustaining HHT concentration in the lungs. The optimal treatment cycles can only be found empirically. Nevertheless, given the *in vitro* and *in vivo* data, the scale of the clinical trial can be delineated. If the scheme works as expected, the nebulization of HHT should show a strong reduction in the viral load of several orders of magnitude within a few days. We estimate that a clinical trial of a modest number of patients (10–50) should be sufficient to prove or disprove the efficacy of HHT.

## FINAL REMARKS

The main obstacle for the HHT clinical trials in many countries is the very low numbers of new COVID-19 patients. At the same time, medical facilities elsewhere may have an acute need for treating new COVID-19 patients by reducing their viral loads. For these facilities, the proposed scheme here may be an option for small-scale clinical trials.
